# Functional Characterization of Novel Atrial Fibrillation-Linked *GJA5* (Cx40) Mutants

**DOI:** 10.3390/ijms19040977

**Published:** 2018-03-25

**Authors:** Mahmoud Noureldin, Honghong Chen, Donglin Bai

**Affiliations:** Department of Physiology and Pharmacology, University of Western Ontario, London, ON, N6A 5C1 Canada; mnourel@uwo.ca (M.N.); hchen38@uwo.ca (H.C.)

**Keywords:** atrial fibrillation, gap junction channel, connexin40, V_j_ gating, patch clamp

## Abstract

Atrial fibrillation (AF) is the most common form of cardiac arrhythmia. Recently, four novel heterozygous Cx40 mutations—K107R, L223M, Q236H, and I257L—were identified in 4 of 310 unrelated AF patients and a followup genetic analysis of the mutant carriers’ families showed that the mutants were present in all the affected members. To study possible alterations associated with these Cx40 mutants, including their cellular localization and gap junction (GJ) function, we expressed GFP-tagged and untagged mutants in connexin-deficient model cells. All four Cx40 mutants showed clustered localization at cell–cell junctions similar to that observed of wildtype Cx40. However, cell pairs expressing Cx40 Q236H, but not the other individual mutants, displayed a significantly lower GJ coupling conductance (G_j_) than wildtype Cx40. Similarly, co-expression of Cx40 Q236H with Cx43 resulted in a significantly lower G_j_. Transjunctional voltage-dependent gating (V_j_ gating) properties were also altered in the GJs formed by Q236H. Reduced GJ function and altered V_j_ gating may play a role in promoting the Q236H carriers to AF.

## 1. Introduction

Atrial fibrillation (AF) is the most common sustained cardiac arrhythmia affecting millions of people worldwide [1,2]. With an overall prevalence of 1%, AF increases with age, starting from 0.1% in individuals younger than 55 years and reaching 9% in those over 80 years [3]. AF prevalence is expected to increase substantially due to an aging population [3]. AF is characterized by a fast sporadic beating of the atria, which causes substantial morbidity including a much higher risk of stroke [2,4]. Often, AF exists as a secondary disease to a wide range of other diseases, such as hypertension, diabetes, and coronary artery disease [5]. However, AF is the primary disease in about 30% of AF patients who are categorized as AF with genetic predisposition [1,6,7]. This group of AF patients has been linked to multiple genetic mutations including genes encoding ion channels, such as potassium channels, sodium channels, and gap junction (GJ) channels [8,9,10,11,12,13,14].

GJ channels are composed of connexins. In humans, there are 21 different connexins and all of them share a similar topological structure of four transmembrane domains (M1–M4), two extracellular domains (E1 and E2), one cytoplasmic loop (CL), and both the amino terminus (NT) and carboxyl terminus (CT) lie within the cytoplasm [15,16]. Six connexins oligomerize to form a hemichannel (also known as connexon) that could function as a channel on the plasma membrane [17]. Two hemichannels from adjacent cells could dock head-to-head at their extracellular domains to form a GJ channel [18]. Human heart expresses three different types of connexins—Cx40, Cx43, and Cx45 [19,20]—allowing for the possible formation of homomeric or heteromeric hemichannels and homotypic or heterotypic GJ channels. Cx45 is dominantly expressed in the sinoatrial (SA) and atrioventricular (AV) nodes while Cx43 and Cx40 are both expressed in the atrial myocardium and are often found to be co-localized at the intercalated discs between atrial myocytes [21,22]. Cx43 is the main connexin in the ventricles [23]. A much lower level of Cx45 is expressed in the atria and ventricles [24]. These connexins form GJ channels between cardiomyocytes to mediate rapid propagation of action potentials (APs) in the heart [24,25].

The importance of Cx40 and Cx43 in the heart has been highlighted in animal models and genetic mutation studies. Mice with an ablation of Cx43 in the heart develop ventricular arrhythmias leading to sudden cardiac death [26]. An in vitro study using cultured atrial synthetic strands from Cx43-deficient mice showed a decrease in conduction velocity [27]. Moreover, an early onset of AF is associated with a somatic Cx43 mutant, which exhibits GJ impairment [28]. Interestingly, viral expression of the exogenous wildtype Cx43 in the atria was found to prevent AF in pig models [29,30]. For Cx40, earlier studies reported that mice lacking the Cx40 gene exhibit a slower action potential propagation [31,32] and are more susceptible to inducible atrial arrhythmias [32,33]. Recent studies reported an increased conduction velocity and a decrease in the conduction heterogeneity in Cx40 knockout mice or cells derived from these mice [27,34,35]. Furthermore, Cx40 promoter polymorphisms result in lower levels of Cx40 mRNA and have been linked to an early onset of AF [36]. Somatic and germline mutations within the coding regions of human Cx40 gene (*GJA5*) have been linked to AF patients and families [11,12,13,14]. A recent genetic study identified four novel germline mutants in *GJA5* in four of 310 unrelated AF patients, resulting in heterozygous missense mutants in Cx40 protein: K107R, L223M, Q236H, and I257L [37]. Further testing on available relatives of the mutant carriers revealed that these mutants presented in all affected family members and were absent in 400 reference alleles [37]. Functional consequences of these AF-linked Cx40 mutants have not been studied. We hypothesize that these AF-linked Cx40 mutants impair GJ and/or hemichannel function, which may predispose the mutant carriers to AF.

## 2. Results

### 2.1. AF-Linked Cx40 Mutants Formed GJ Plaque-Like Structures at the Cell–Cell Interface

**Figure 1:**
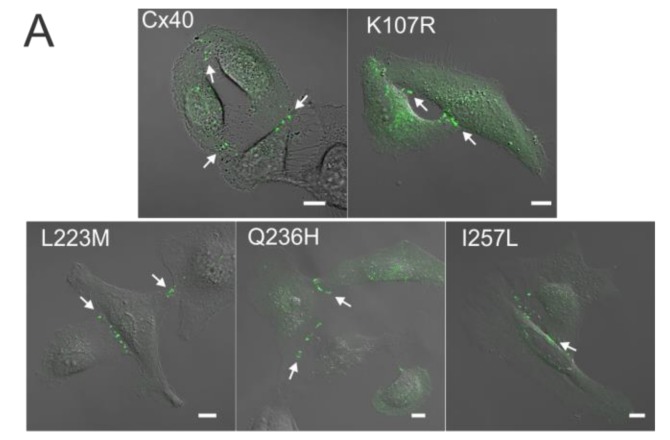

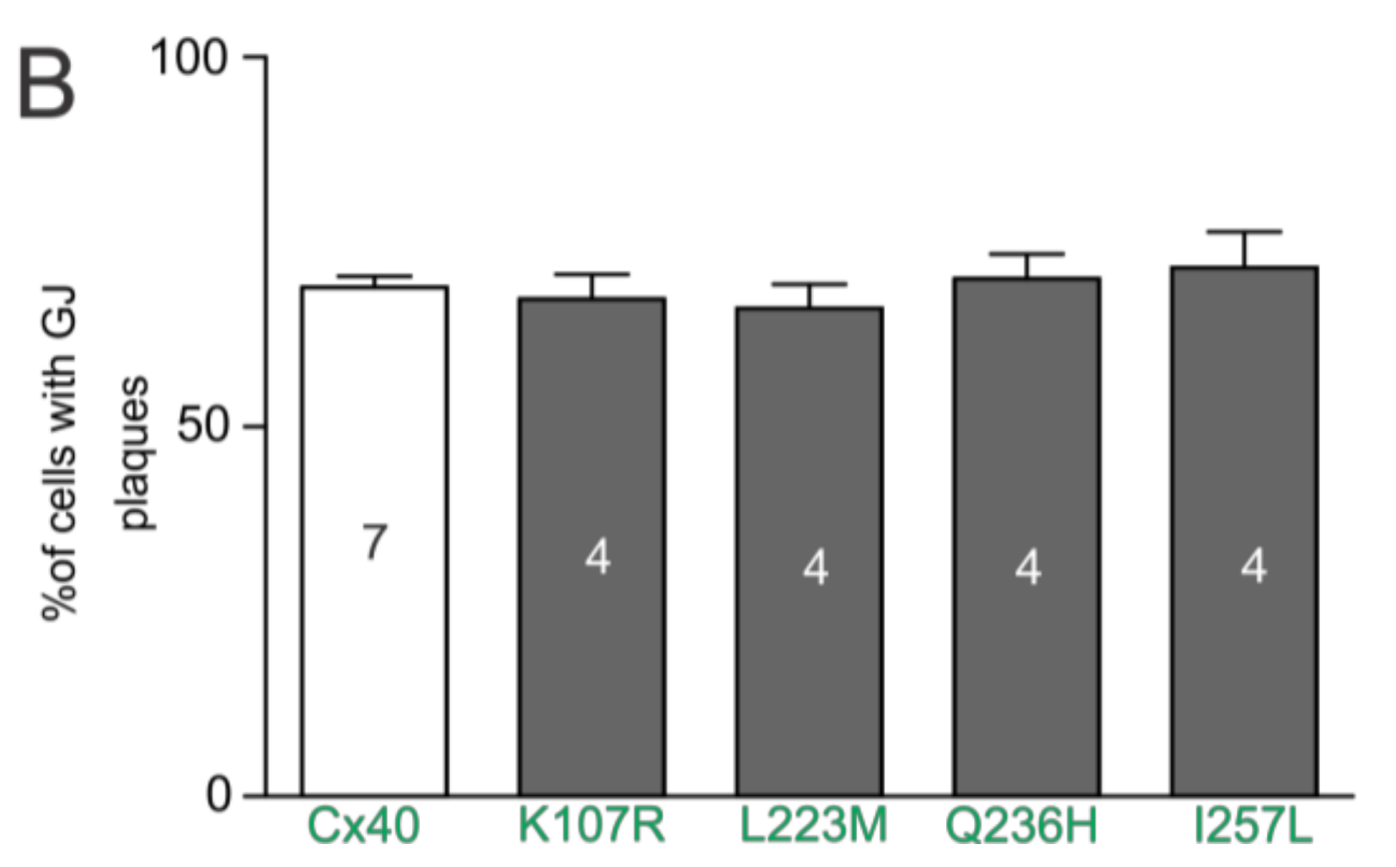
Localization of atrial fibrillation (AF)-linked Cx40 mutants. (**A**) Fluorescent images of HeLa cell clusters or pairs expressing YFP-tagged Cx40, K107R, L223M, Q236H, or I257L superimposed on their respective differential interference contrast (DIC) images. Cells expressing each of the AF-linked Cx40 mutants were able to form GJ plaque-like structures at the cell–cell interface similar to that of wildtype Cx40 (white arrows). Scale bars = 10 µm; (**B**) the bar graph summarizes the percentage of cell pairs showing GJ plaque-like structures at the cell–cell interface for each mutant. No statistical difference was observed between any of the mutants and wildtype Cx40. Approximately 100 positively-transfected cell pairs were examined for each transfection. The total number of transfections is indicated on each bar.

Expression of AF-linked Cx40 mutants (K107R, L223M, Q236H, and I257L, all tagged with YFP at the carboxyl terminus) was used to study their localization in live HeLa cells. As shown in Figure 1A, each of the mutants was localized in intracellular compartments and displayed GJ plaque-like clusters at the cell–cell interfaces similar to that of Cx40–YFP. The percentage of successful mutant-expressing cell pairs displaying GJ plaque-like structures at cell–cell interfaces was calculated and was found to be similar to that of cells expressing wildtype Cx40 (Figure 1B).

### 2.2. Coupling Conductance of GJs Formed by AF-Linked Mutants

**Figure 2:**
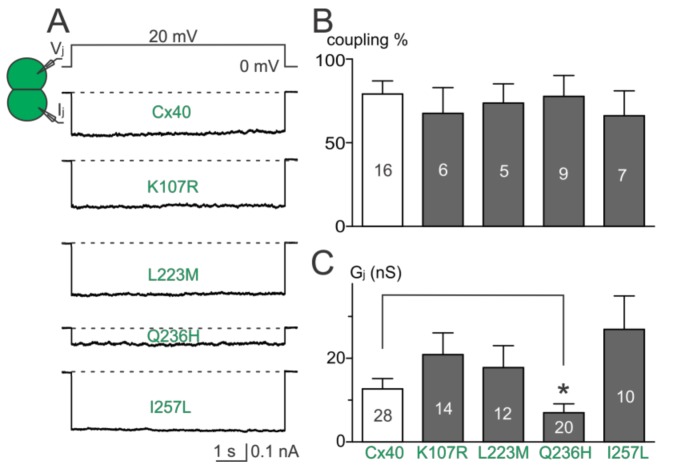
Coupling percentage and G_j_ of AF-linked mutants. (**A**) Dual whole-cell patch clamp technique was used to measure junctional current (I_j_) from N2A cell pairs expressing untagged Cx40, K107R, L223M, Q236H, or I257L at 20 mV V_j_; (**B**) bar graph summarizes the coupling percentages of cell pairs expressing the AF-linked Cx40 mutants. No statistical difference was observed between each of the mutants and the wildtype Cx40. The number of transfections is indicated on each bar; (**C**) bar graph illustrates the coupling conductance (G_j_) of coupled cell pairs expressing Cx40, K107R, L223M, Q236H, or I257L. Cell pairs expressing Q236H showed a significantly lower G_j_ than those of wildtype Cx40 (* *p* < 0.05). The number of cell pairs is indicated on each bar.

Dual whole-cell patch clamp was used to study the functionality of untagged AF-linked Cx40 mutants in N2A cell pairs. Representative junctional currents (I_j_s) of cell pairs expressing each of the Cx40 mutants and wildtype Cx40 are presented (Figure 2A). The averaged coupling percentage of each Cx40 mutant in several transfections, plotted as a bar graph, was not different from that of wildtype Cx40 (Figure 2B, *p* > 0.05 for each of the mutants). The coupling conductance (G_j_) of cell pairs expressing K107R, L223M, or I257L was also not different from that of wildtype Cx40 (Figure 2C). However, a significant reduction in G_j_ was observed in cell pairs expressing Q236H (Figure 2C, *p* < 0.05).

### 2.3. Homotypic Cx40 Q236H GJs Showed an Altered V_j_ Gating

**Figure 3:**
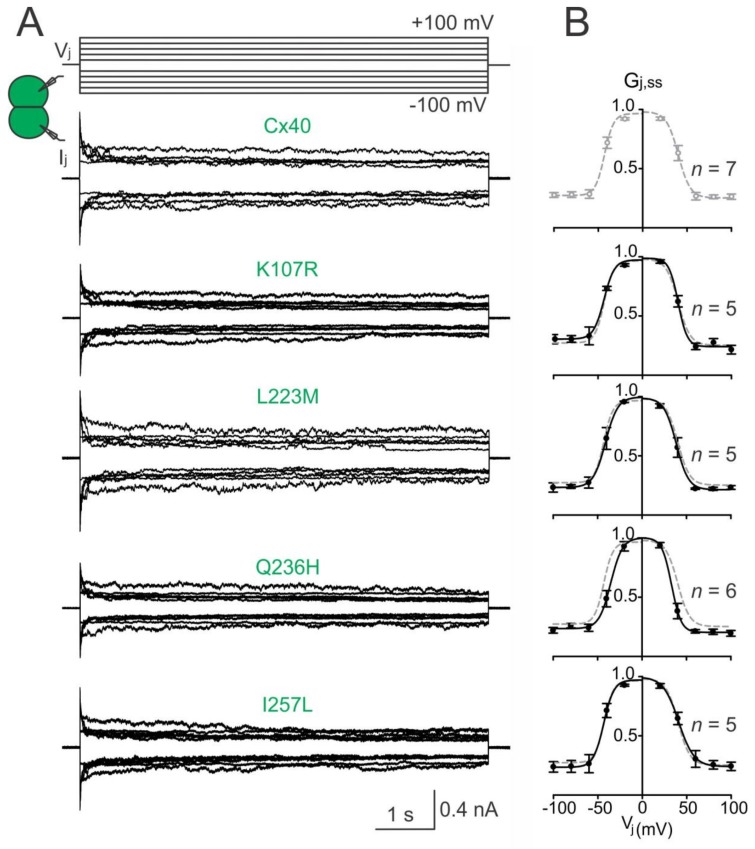
V_j_ gating of AF-linked mutant GJs. (**A**) Dual whole-cell patch clamp was used to measure I_j_s in N2A cell pairs expressing Cx40, K107R, L223M, Q236H, or I257L in response to a series of V_j_ pulses as indicated. Superimposed I_j_s for each mutant is shown; (**B**) normalized steady state junctional conductance, G_j,ss_, of the Cx40 mutants (black filled circles) and wildtype Cx40 (grey open circles) were plotted at different V_j_s. The G_j,ss_–V_j_ plot of each mutant was fitted with a two-state Boltzmann equation at each V_j_ polarity (smooth black lines). Boltzmann fittings of G_j,ss_–V_j_ plot of wildtype Cx40 (smooth grey dashed lines) were obtained and superimposed on each plot for comparison. The number of cell pairs is indicated.

To investigate the transjunctional voltage-dependent gating (V_j_ gating) of AF-linked Cx40 mutants, we measured I_j_s in cell pairs in response to a series of V_j_ pulses (±20 to ±100 mV, Figure 3A). The I_j_s from cell pairs expressing untagged Cx40 mutant K107R, L233M, Q236H, or I257L showed similar symmetrical V_j_-dependent deactivation (sometimes also called inactivation) when V_j_s ≥ 40 mV (Figure 3). The normalized steady state conductance (G_j,ss_) of each mutant (filled circles) or wildtype Cx40 (open grey circles) was plotted at different V_j_s (Figure 3B). The smooth black lines are Boltzmann fitting curves for each of the mutants (Figure 3B). Boltzmann fittings of wildtype Cx40 (smooth grey dashed lines) are plotted and superimposed onto each mutant G_j,ss_–V_j_ plot for comparison (Figure 3B). Compared to the wildtype Cx40, GJ channels formed by these mutants showed nearly identical Boltzmann fitting curves, except Q236H, which showed a significant reduction in V_0_ for both V_j_ polarities (Figure 3B, Table 1).

**Figure 4:**
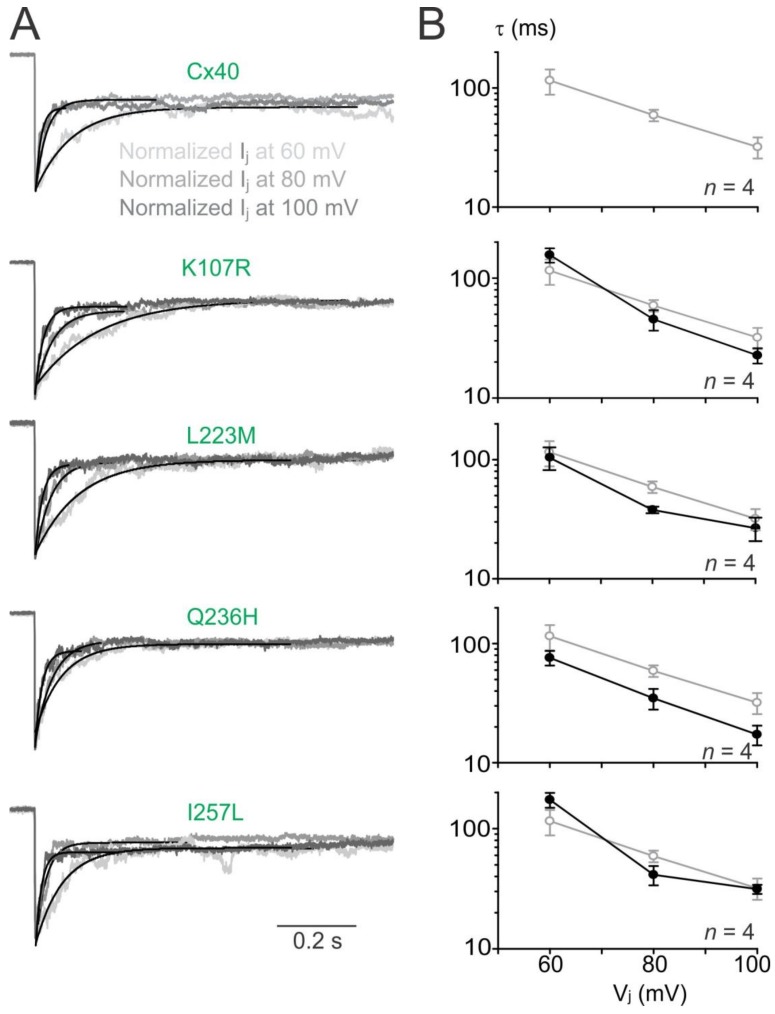
V_j_-gating kinetics of AF-linked Cx40 mutants. (**A**) I_j_s induced at different V_j_s (60 mV light grey, 80 mV medium grey, 100 mV dark grey) were normalized and superimposed for each of the mutant or Cx40 GJs. I_j_ deactivations under different V_j_s were all fitted well with a single exponential process (smooth black lines); (**B**) The time constants (τs) were plotted on a semi logarithmic scale against different V_j_s. When the V_j_s increased, the averaged τs of the mutant GJs (black filled circles) decreased similar to those observed for the wildtype Cx40 (grey open circles). No consistent statistical difference was found between most of the mutant τs and the τs of wildtype Cx40, except the τs of Q236H was consistently lower than those of wildtype Cx40 (two-way ANOVA). The number of cell pairs are indicated.

To analyze V_j_-gating kinetics, we fitted the I_j_ deactivation by a single exponential process at V_j_s of ±60 to ±100 mV. As shown in Figure 4A, I_j_ deactivation of wildtype Cx40 GJs fitted well with a single exponential process (with a time constant, τ) at each of the tested V_j_s (Figure 4A). The averaged time constants (τs) showed a decrease with the increase of V_j_s (Figure 4B, open grey circles). The I_j_ deactivations of the GJs formed by AF-linked mutants could be fitted by a single exponential process and the τ–V_j_ plots were not statistically different from those of wildtype Cx40 GJs (filled black circles), except Q236H GJ that showed consistently lower τs at all tested V_j_s (Figure 4B, *p* < 0.05).

### 2.4. Co-Expression of AF-Linked Mutants with Cx43

**Figure 5:**
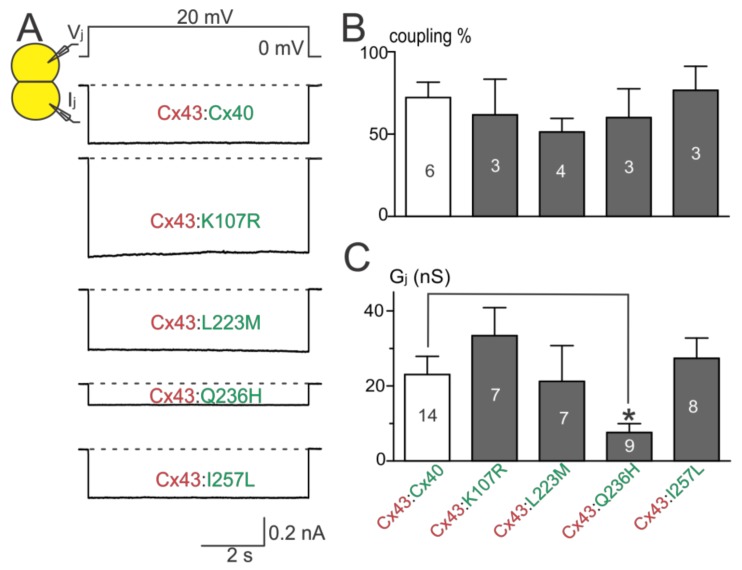
Coupling percentage and G_j_ of co-expressing AF-linked mutants with wildtype Cx43. (**A**) Representative I_j_s are shown from N2A cell pairs co-expressing Cx40, K107R, L223M, Q236H, or I257L (with an untagged reporter GFP) with wildtype Cx43 (with an untagged reporter DsRed); (**B**) bar graph illustrates coupling percentages of N2A cell pairs expressing each combination. The number of transfections is indicated; (**C**) bar graph illustrates the G_j_ of cell pairs co-expressing one of the Cx40 mutants (K107R, L223M, Q236H, or I257L) with Cx43. The G_j_ of cell pairs co-expressing Q236H:Cx43 was significantly lower than that of wildtype Cx40:Cx43 (* *p* < 0.05). The number of cell pairs is indicated.

To investigate if AF-linked Cx40 mutants had a trans-dominant negative effect on wildtype Cx43, each of the mutants was co-expressed with Cx43 (with an untagged DsRed). Cell pairs successfully expressing both connexins were selected for dual whole-cell patch clamp. Cell pairs successfully co-expressing K107R:Cx43, L223M:Cx43, or I257L:Cx43 showed coupling percentages and G_j_s that were not statistically different from those of wildtype Cx40:Cx43 (Figure 5B,C). The coupling percentage of Q236H:Cx43 was also not statistically different from that of wildtype Cx40:Cx43. However, the G_j_ of cell pairs co-expressing Q236H:Cx43 was significantly lower than that of wildtype Cx40:Cx43 (Figure 5C, *p* < 0.05).

### 2.5. Function of Heterotypic Mutant/Cx40 GJ Channels

**Figure 6:**
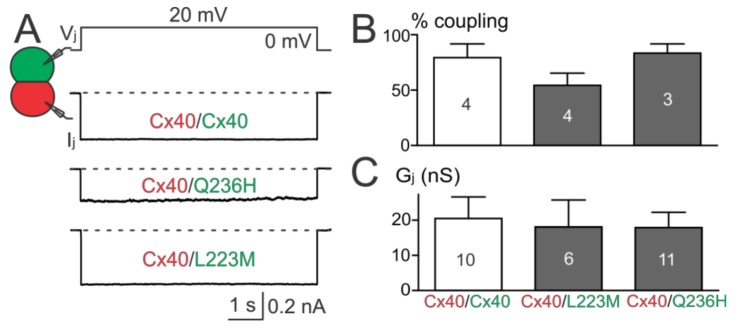
Functional test on heterotypic mutant/Cx40 GJs. (**A**) I_j_s were obtained from heterotypic Q236H/Cx40, L223M/Cx40, or Cx40/Cx40 (in all cases with untagged GFP or DsRed, respectively) N2A cell pairs; (**B**) Bar graph summarizes the coupling percentages of heterotypic cell pairs. No statistical difference was found between the coupling percentage of any of the mutant heterotypic GJs and that of Cx40/Cx40 GJs. The number of transfections is indicated; (**C**) G_j_s of cell pairs expressing L223M/Cx40 or Q236H/Cx40 were not statistically different from those of Cx40/Cx40. The number of cell pairs is indicated.

The above results showed that Cx40 Q236H had a significantly lower G_j_s than wildtype Cx40 when expressed alone or co-expressed with Cx43. To further test whether Q236H could affect the function of heterotypic Q236H/Cx40 GJs, we mixed cells expressing Q236H (with untagged GFP) with cells expressing Cx40 (with untagged DsRed) and performed dual patch clamp on heterotypic cell pairs (one GFP+ and the other DsRed+, Figure 6A). The coupling percentages and G_j_s of heterotypic Q236H/Cx40 cell pairs were not statistically different from those of the control (Cx40/Cx40, Figure 6B,C). Similar results were also obtained for L223M (Figure 6).

### 2.6. Propidium Iodide Uptake by AF-Linked Cx40 Mutant-Expressing Cells

**Figure 7:**
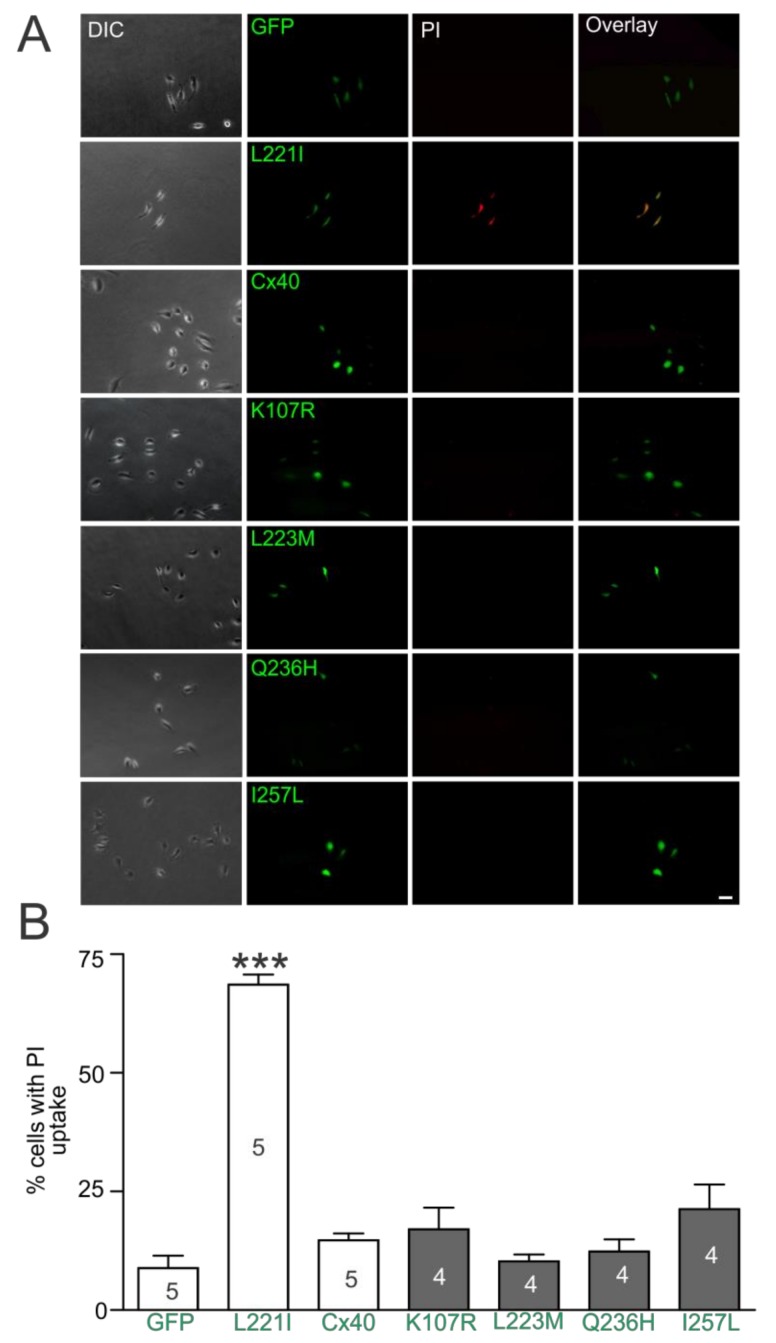
Propidium iodide uptake of AF-linked Cx40 mutants. (**A**) HeLa cells transfected with Cx40 mutants, empty vector GFP, or Cx40 are shown: column 1 (under DIC), column 2 (GFP fluorescence to show successful expression of respective vector), column 3 (propidium iodide [PI] uptake in red), column 4 (an overlay of images of column2 and 3). Only cells expressing L221I showed PI uptake. The scale bar = 50 μm; (**B**) bar graph summarizes PI uptake percentage of isolated individual cells expressing Cx40 mutants, Cx40, or GFP. PI uptake percentage for each of the AF-linked mutants was not statistically different from that of wildtype Cx40 or the empty vector (GFP), except L221I (*** *p* < 0.001). The number transfection is indicated with observations of over 60 isolated cells for each transfection.

Propidium iodide (PI) uptake assay was used to investigate the hemichannel function of AF-linked Cx40 mutants as elevated PI uptake was observed in AF-linked Cx40 mutants, including L221I [38,39]. Figure 7A shows the fluorescent images of individual HeLa cells expressing GFP alone, Cx40, or one of the mutants (L221I, K107R, L223M, Q236H, or I257L) in divalent cation-free solution. The percentage of individual cells showing PI uptake in cells expressing K107R, L223M, Q236H or I257L was not significantly different from either the wildtype Cx40 or the negative control (expressing GFP alone) but was statistically lower than the positive control L221I (69%, *p* < 0.001) (Figure 7B). These results suggest that the Cx40 mutants and the wildtype Cx40 failed to show PI uptake in divalent cation-free solution.

## 3. Discussion

In this study, we examined morphological and functional characteristics of four recently identified AF-linked Cx40 mutations (K107R, L223M, Q236H and I257L) in vitro. Our localization experiments showed that YFP-tagged K107R, L223M, Q236H, and I257L were able to form GJ plaque-like structures at the cell–cell interface in HeLa cells, similar to that of wildtype Cx40. PI uptake by each of these mutant (untagged)-expressing cells showed no significant increase from that of Cx40, indicating no increase in hemichannel activity in any of the mutant-expressing cells. Dual patch clamp experiments revealed that the Cx40 mutants (K107R, L223M, and I257L) showed no apparent change in coupling conductance (G_j_) when expressed alone or together with Cx43. The GJs formed by each of these mutants also failed to show any obvious change in the V_j_ gating properties. In contrast, Q236H GJs exhibited a significantly reduced G_j_ when expressed alone or together with Cx43. In addition, Q236H GJs also showed altered V_j_ gating, specifically a reduction in the V_j_ required to close the channel (V_0_) and an increase in V_j_-gating kinetics. These defects associated with Q236H might play a role in the pathogenesis of AF in the mutant carriers.

### 3.1. AF-Linked Cx40 Mutants Showed Multiple Defects in GJ or Hemichannel Function

So far, a total of ten germlines and three somatic mutations in the coding region of the *GJA5* gene (encoding Cx40) have been identified in AF patients with genetic predisposition [11,12,13,14,37,40]. In vitro studies on these AF-linked Cx40 mutants have revealed that these mutants display either a loss of GJ function or a gain of hemichannel function. The detailed molecular and cellular mechanisms leading to GJ or hemichannel functional changes appear to be quite different. (1) A Cx40 missense mutation P88S failed to localize to cell–cell interfaces to form GJ plaque-like structures [11]. Similarly, a nonsense Cx40 mutation (Q49X) was found to be retained in the endoplasmic reticulum and unable to reach cell–cell junctions [41]. Functional impairment of GJs in these two mutants were anticipated and confirmed experimentally, but interestingly both mutants showed dominant negative and transdominant negative actions on GJ function when co-expressed with wildtype Cx40 or Cx43, respectively [11,41]. (2) Other AF-linked Cx40 missense mutants (G38D, I75F, V85I, A96S, M163V, L221I, L229M) and those in the present study (K107R, L223M, Q236H, and I257L) showed GJ plaques at the cell–cell interfaces [11,14,38,39]. However, eliminated or significantly reduced macroscopic coupling conductance (G_j_) was observed in G38D, I75F, A96S, and Q236H GJs, probably due to impairment at the GJ channel [11,42]. Some of the mutants in this category were also found to show dominant negative action on Cx40 (I75F and A96S) and/or transdominant negative action on Cx43 (I75F, A96S, L229M, and Q236H) [11,42]. Although some isolated disagreements on the G_j_ levels of G38D and A96S GJs have been reported [39,43], the majority of these studies agree on the GJ functional impairments in most of the AF-linked Cx40 mutants [11,14,39,41,43]. (3) Detailed characterizations of mutant-containing GJ channels revealed additional defects, including reduced G_j_ of heterotypic mutant/wildtype GJs, altered V_j_ gating properties of homotypic (G38D, A96S, M163V, Q236H) or heterotypic (I75F/Cx40, A96S/Cx40) V_j_ gating properties, a substantially reduced open probability without changing unitary channel conductance (I75F), or elevated unitary channel conductance (G38D and M163V) [14,39,42,43]. (4) Only a limited number of AF-linked mutants (G38D, M163V, and A96S) have been studied for GJ permeability changes but significant permeability change to anionic dye (Lucifer yellow) or cationic dye (ethidium bromide) was observed [43]. (5) The PI uptake assay was used to study hemichannel function in isolated cells expressing AF-linked mutants. Among the tested Cx40 mutants, only V85I- and L221I-expressing cells showed an elevated PI uptake compared to that of wildtype Cx40, indicating a gain of hemichannel function in these mutants [38]. Patch clamp on cells expressing Cx40 G38D showed unitary hemichannel currents [39]. Similar elevated hemichannel function was also observed in a few other disease-linked mutants in Cx26, Cx43, and Cx50 [44,45,46,47].

### 3.2. AF-Linked Cx40 Mutants and Their Possible Role in AF Pathogenesis

As discussed above, there is a variety of molecular/cellular changes associated with AF-linked Cx40 mutants. Whether these molecular/cellular changes play a role in the pathogenesis of AF is not clear. Several theoretical possibilities exist. First, a reduced macroscopic coupling conductance (G_j_) of AF-linked Cx40 mutants due to either an impaired localization or GJ channel function is known to reduce the action potential conduction velocity [48,49], which could be an important contributing factor in promoting re-entrant atrial arrhythmias [50]. Consistent with this model, about half of AF-linked Cx40 mutants identified so far have shown G_j_ reduction not only in the mutant GJs but also when they are co-expressed with wildtype Cx40 and/or Cx43 [51]. Our present study showed that Cx40 Q236H also reduced G_j_ when expressed alone and together with Cx43. We did not perform co-expression of this mutant with wildtype Cx40 because our untagged Cx40 construct (Cx40-IRES-DsRed) had a very low transfection efficiency. Second, enhanced V_j_ gating by lower V_j_s and faster gating kinetics by Cx40 mutants could also dynamically down-regulate G_j_ when sufficient junctional delays exist [52,53]. Cx40 showed a pronounced V_j_ gating with a minimum conductance level reaching a quarter of the maximum G_j_ [54,55]. A reduction in the Boltzmann fitting parameter, V_0_, and faster V_j_-dependent deactivation kinetics of Q236H mutant GJs predict an increased V_j_ gating when sufficient junctional delay exists. It is not clear whether V_j_ gating of Cx40 or Q236H GJs could dynamically down-regulate G_j_ as observed for Cx45 GJs [56]. Third, AF-linked Cx40 mutants have been shown to alter their GJ permeability [43], which could alter intercellular exchanges of small signaling molecules, including second messengers. This altered permeability of GJs could restrict/enhance signaling molecules necessary for intercellular communication between atrial myocytes, which might be important for atrial function. Fourth, three of the AF-linked mutants showed elevated PI uptake and/or hemichannel current, indicating enhanced hemichannel activity under reduced divalent cations in the extracellular medium [38,39]. Physiological and/or pathological stresses, such as large repetitive membrane depolarizations, mechanical stretch, reduced extracellular divalent cations, reduced oxygen/glucose during ischemia, have all been shown to enhance several other connexin hemichannels. Whether these stress factors also promote the opening of Cx40 hemichannel remains to be determined. In summary, it is not clear how these defects in Cx40 mutants in model cells link to the pathogenesis of AF in the mutant carriers. Among the changes associated with AF-linked Cx40 mutants, the most consistent is a reduced or eliminated GJ coupling (G_j_) in different possible atrial GJs. At present, we cannot rule out other changes, such as biosynthesis, and turnover of the mutant Cx40 protein may also change the abundance and function of Cx40 at the intercalated discs, which may need genetically modified animal models to assess fully.

### 3.3. AF-Linked Cx40 Mutants without Apparent Defects In Vitro

In our present study, we did not detect any obvious defects in GJ distribution, function, or hemichannel activities in three out of four AF-linked Cx40 mutants (K107R, L223M, and I257L). It is not clear how these mutants relate to AF. Here are some possibilities. (1) These mutants are located in the CL (K107R), M4 (L223M), and CT (I257L) domains of Cx40. The CL and CT domains of Cx40 show a lot more residue variation in different vertebrate species than the E1, E2, and M1–4 domains. Following this general trend, the conservation percentage at these residue positions in Cx40 are L223 (in M4 domain) 85%, K107 (in CL domain) 74%, and I257 (in CT domain) 28% across 47 different species (accessible online: https://omabrowser.org/oma/home/ OMA Group 752281). It is possible that one or more of these mutants are benign mutants that do not necessarily play a role in promoting AF, especially at the least conserved position, I257. (2) Both of our model cells, HeLa and N2A cells, are convenient model systems to study localization, function of GJs, and hemichannels. They are GJ deficient, easily transfected with connexin mutant constructs, and easily accessible for morphological, dual patch clamp, or dye-uptake experiments. However, these cells are not cardiomyocytes and may not recapitulate all aspects of GJs at the intercalated discs of cardiomyocytes and, therefore, some defects might go undetected. Future studies on cell models that are closer to atrial myocytes will likely help to resolve the role of these Cx40 mutants linking to AF. (3) We have good rationales to focus our study on the morphology and functional changes in GJs and hemichannel activities. However, there are other unconventional functions of connexins, including but not limited to aggregation of protein complexes at the cell–cell junctions, adhesion, cell growth control, and differentiation, etc., that require specific biological assays to evaluate.

### 3.4. Other AF-Linked Genetic Factors

It is well known that genetic factors play a role in AF. A lot of research is focused on genes responsible for inherited AF cases as these genes are putative independent AF risk factors [7,57]. The first genetic mutation linked to familial AF was identified in the *KCNQ1* gene, which encodes a potassium channel subunit [9]. Since then, more AF-linked mutations in different potassium channel subunits have been identified and are now extended into genes encoding sodium channels, transcription factors, Ca^2+^ handling proteins, nucleoporins, and atrial natriuretic peptide [8,58]. Our present study is consistent with several previous studies showing that atrial GJ impairments represent an independent risk factor for AF [11,14,28,38,40,41,51].

## 4. Materials and Methods

### 4.1. Plasmid Construction

The C-terminal fusion fluorescent protein tagged human Cx40-YFP and the untagged constructs (Cx40-IRES-GFP, Cx43-IRES-DsRed, Cx40-IRES-DsRed) were created as previously described [14,59]. The novel AF-linked tagged and untagged Cx40 mutants were generated by site-directed mutagenesis on the corresponding tagged/untagged Cx40 as templates with the following primers.

K107RForward: 5′ CAGGAGAAGCGCAGGCTACGGGAGGCC 3′Reverse: 5′ GGCCTCCCGTAGCCTGCGCTTCTCCTG 3′L223MForward: 5′ CTCCTCCTTAGCATGGCTGAACTCT 3′Reverse: 5′ AGAGTTCAGCCATGCTAAGGAGG 3′I257LForward: 5′ CCCTCTGTGGGCCTAGTCCAGAGCTGC3′Reverse: 5′ GCAGCTCTGGACTAGGCCCACAGAGGG3′Q236HForward: 5′ GGAAGAAGATCAGACACCGATTTGTCAAACC3′Reverse: 5′ GGTTTGACAAATCGGTGTCTGATCTTCTTCC3′

All these Cx40 mutant constructs were sequenced to confirm the accuracy of the nucleotide sequence.

### 4.2. Cell Culture and Transfection

Connexin-deficient mouse neuroblastoma (N2A) and human cervical carcinoma (HeLa) cells (American Type Culture Collection, Manassas, VA, USA) were cultured at 37 °C with 5% CO_2_. Cells were grown in Dulbecco’s modified Eagle’s medium (DMEM) (Cat# 10313-021, Thermo Fisher Scientific, Waltham, MA, USA) containing 10% fetal bovine serum, 1% penicillin, 1% streptomycin, 4.5 g/L d-(+)-glucose, 584 mg/L l-glutamine, and 110 mg/L sodium pyruvate. Twenty-four hours before cell transfection, N2A or HeLa cells were replated into a 35-mm dish at 60% confluency. Transfection was performed the next day by adding 0.8–1 μg of DNA with 2 μL of the transfection reagent X-tremeGENE HP DNA (Roche Applied Sciences, Indianapolis, IN, USA). To assess the effect of Cx40 mutants on wildtype Cx43, N2A cells were transfected in a 1:1 ratio of Cx40 mutants-IRES-GFP and Cx43-IRES-DsRed. Cell pairs successfully co-expressing both GFP and DsRed were selected for measuring coupling conductance with dual whole-cell patch clamp (see below).

### 4.3. Localization

HeLa cells were transiently transfected with YFP-tagged Cx40 mutants. One day after transfection, cells were replated on 10 mm glass coverslips and incubated overnight. The number of successfully transfected cell pairs forming GJ plaque-like structures at the cell–cell interface were counted. A confocal microscope (Zeiss LSM800 with Airyscan) (Zeiss, Oberkochen, Germany) was used to observe mutant-YFP and wildtype Cx40-YFP localizations as described earlier [59].

### 4.4. Electrophysiology

On the experimental day, transfected N2A cells were replated onto glass coverslips and incubated for 1.5 to 3 h. Coverslips with cells were transferred into a recording chamber and bathed in extracellular solution (ECS) containing 135 mM NaCl, 5 mM KCl, 10 mM Hepes, 1 mM MgCl_2_, 2 mM CaCl_2_, 1 mM BaCl_2_, 2 mM CsCl, 2 mM Na Pyruvate, and 5 mM d-glucose with pH and osmolarity of 7.4 and 310–320 mOsm, respectively. The recording chamber was placed on an upright fluorescent microscope (BX51WI, Olympus, Center Valley, PA, USA) to visualize reporter (GFP)-positive cell pairs. Patch pipette was filled with intracellular solution (ICS) containing 130 mM CsCl, 10 mM EGTA, 0.5 mM CaCl2, 4 mM Na2ATP, and 10 mM Hepes with pH 7.2 and osmolarity of 290–300 mOsm. Dual whole-cell patch clamp technique was performed on isolated cell pairs expressing the Cx40 mutant. Initially, cell pairs were both voltage clamped at 0 mV. Then, a 20 mV voltage pulse was applied to one of the cell pairs (pulsing cell) while keeping the other clamped at 0 mV (the recording cell). If functional GJ channels exist between the cell pairs then a transjunctional current (I_j_) can be measured at the recording cell via MultiClamp 700A (Molecular Devices, Sunnyvale, CA, USA) and stored in a PC via an AD/DA interface (Digidata 1322A) and pClamp9.2 software (Molecular Devices, Sunnyvale, CA, USA). G_j_ was calculated (G_j_ = I_j_/V_j_). V_j_ gating properties were studied by applying a series of voltage pulses (±20 to ±100 mV in 20 mV increment) as described in our previous studies [14,56].

### 4.5. Dye Uptake Assay

AF-linked Cx40 mutants hemichannel function were assessed using propidium iodide (PI) uptake assay. HeLa cells were transiently transfected with each of the Cx40 mutants in an IRES-GFP vector [38]. We used a previously characterized AF-linked Cx40 mutant L221I-IRES-GFP as the positive control and empty IRES-GFP vector as the negative control for these experiments [38]. Divalent cation containing extracellular solution (DCC-ECS) was composed of 142 mM NaCl, 5.4 mM KCl, 1.4 mM MgCl_2_, 2 mM CaCl_2_, 10 mM HEPES, and 25 mM d-(+)-glucose adjusted to pH 7.35 and osmolarity of ~298 mOsm. HeLa cells were incubated in a divalent cation-free extracellular solution (DCF-ECS) containing PI (150 μM). Removal of both Ca^2+^ and Mg^2+^ ions, as well as including EGTA (2 mM) in the DCF-ECS, were used to facilitate PI uptake via GJ hemichannels. After incubation at 37 °C for 15 min, cells were washed three times with DCC-ECS at room temperature prior to observation with a fluorescent microscope (DMIRE2, Leica, Cridersville, OH, USA). The number of transfected HeLa cells with or without PI uptake was counted and the percentage of cells with PI uptake was calculated. Only isolated individual HeLa cells were counted to prevent errors caused by GJ channels in cell clusters.

### 4.6. Data Analysis

Mann–Whitney U test was used to compare each of the mutants against wildtype Cx40 for the percentage of cell pairs with morphological GJ plaques, the coupling percentage and conductance (G_j_) using dual patch clamp, and the percentage of cells displaying PI uptake for the hemichannel study. One-way ANOVA followed by Tukey post-hoc test was used to compare the Boltzmann fitting parameters of each mutant and wildtype Cx40 at the corresponding V_j_ polarity. Statistical significance is denoted with different levels of significance (* *p* < 0.05; ** *p* < 0.01; or *** *p* < 0.001).

## 5. Conclusions

In summary, our results indicate that the AF-linked Cx40 Q236H mutation exhibited GJ function impairment by reducing the overall G_j_ when expressed alone or with the wildtype Cx43, and altered V_j_-gating kinetics and the V_0_, which might play a role in AF pathogenesis. The other mutants—K107R, L223M, and I257L—did not exhibit any apparent GJ or hemichannel functional impairments in our cell models.

## Figures and Tables

**Table 1:** Boltzmann fitting parameters for V_j_ gating of AF-linked mutants.

Cells Expressing	V_j_ Polarity	G_min_	V_0_ (mV)	*A*
Cx40 (*n* = 7)	+	0.25 ± 0.02	40.2 ± 1.4	0.15 ± 0.05
	−	0.27 ± 0.02	42.9 ± 1.4	0.19 ± 0.07
K107R (*n* = 5)	+	0.21 ± 0.03	38.6 ± 1.6	0.15 ± 0.05
	−	0.23 ± 0.03	41.1 ± 1.9	0.15 ± 0.05
L223M (*n* = 5)	+	0.24 ± 0.02	40.2± 1.0	0.20 ± 0.10
	−	0.31 ± 0.03	43.2 ± 1.8	0.17 ± 0.06
Q236H (*n* = 6)	+	0.20 ± 0.02	33.3 ± 1.7 *^,1^	0.19 ± 0.04
	−	0.24 ± 0.02	35.2 ± 2.0 *	0.15 ± 0.04
I257L (*n* = 5)	+	0.24 ± 0.03	40.9 ± 2.2	0.12 ± 0.03
	−	0.24 ± 0.03	43.4 ± 2.2	0.17 ± 0.08

^1^ One-way ANOVA followed by Tukey *post-hoc* test was used to compare the Boltzmann fitting parameters of each mutant and wildtype Cx40 at the corresponding V_j_ polarity. GJ channels formed by the mutant Q236H showed a significantly lower V_0_ for both V_j_ polarities than those of wildtype Cx40 (* *p* < 0.05).

## Abbreviations

Cx40	connexin40
G_j,ss_	normalized steady-state junction conductance
I_j_	macroscopic junctional current
V_j_	transjunctional voltage
